# Combined inhibition of CDK and HDAC as a promising therapeutic strategy for both cutaneous and uveal metastatic melanoma

**DOI:** 10.18632/oncotarget.23485

**Published:** 2017-12-15

**Authors:** Renier Heijkants, Karen Willekens, Mark Schoonderwoerd, Amina Teunisse, Maaike Nieveen, Enrico Radaelli, Luuk Hawinkels, Jean-Christophe Marine, Aart Jochemsen

**Affiliations:** ^1^ Department of Molecular Cell Biology, Leiden University Medical Center, Leiden, The Netherlands; ^2^ Laboratory for Molecular Cancer Biology, VIB Center for Cancer Biology, Leuven, Belgium; ^3^ Department of Oncology, KU Leuven, Leuven, Belgium; ^4^ Department of Gastroenterology-Hepatology, Leiden University Medical Center, Leiden, The Netherlands; ^5^ Mouse Histopathology Core Facility, VIB Center for the Biology of Disease, KU Leuven, Leuven, Belgium

**Keywords:** metastasized melanoma, CDK, HDAC, apoptosis, synergism

## Abstract

Very little to no improvement in overall survival has been seen in patients with advanced non-resectable cutaneous melanoma or metastatic uveal melanoma in decades, highlighting the need for novel therapeutic options. In this study we investigated as a potential novel therapeutic intervention for both cutaneous and uveal melanoma patients a combination of the broad spectrum HDAC inhibitor quisinostat and pan-CDK inhibitor flavopiridol. Both drugs are currently in clinical trials reducing time from bench to bedside. Combining quisinostat and flavopiridol shows a synergistic reduction in cell viability of all melanoma cell lines tested, irrespective of their driver mutations. This synergism was also observed in BRAF^V600E^ mutant melanoma that had acquired resistance to BRAF inhibition. Mechanistically, loss of cell viability was, at least partly, due to induction of apoptotic cell death. The combination was also effectively inducing tumor regression in a preclinical setting, namely a patient-derived tumor xenograft (PDX) model of cutaneous melanoma, without increasing adverse effects. We propose that the quisinostat/flavopiridol combination is a promising therapeutic option for both cutaneous and uveal metastatic melanoma patients, independent of their mutational status or (acquired) resistance to BRAF inhibition.

## INTRODUCTION

Melanoma is an aggressive type of cancer which originates from melanocytes, affecting about 132,000 new patients in 2016 in the US alone [1]. Although melanoma is found predominantly as a cutaneous disease, melanomas from the uveal tract in the eye, uveal melanoma (UM), account for ∼5.3% of total melanoma incidence [2]. UM is genetically distinct from cutaneous melanoma (CM). CM is most commonly driven by oncogenic mutations in NRAS or BRAF [3]; the latter spurred the development of mutant-specific BRAF inhibitors. Although most patients with BRAF mutations initially respond well to BRAF inhibition, resistance and relapse inevitably occurs within 6 to 8 months [4]. Besides BRAF inhibitors, immunotherapy has proven to be an effective treatment in CM cases [5]. In contrast, UM is in most cases driven by an activating mutation in one of the G-proteins GNA11 or GNAQ [6, 7]. It has been shown that the continuous activation GNA11 or GNAQ exerts its oncogenic capacity, among others, through the activation of the MAPK pathway via protein kinase C (PKC) signaling [8–10]. This insight has incited the use of PKC inhibitors as treatment for UM, but these inhibitors only have limited clinical effects [11]. Despite these ongoing developments there still is a lack of curative treatment for metastasized UM and CM, rendering metastasized melanoma a lethal disease. Our effort to search for novel therapeutic interventions for metastatic melanoma focuses on drugs in clinical development to reduce the time from bench to bedside.

A number of studies have shown promising results using histone deacetylase (HDAC) inhibitors, both in pre-clinical studies and clinical trials, as potential therapeutic intervention for both CM and UM [12–15]. One of these HDAC inhibitors is quisinostat (also known as JNJ-26481585), a second generation broad spectrum HDAC inhibitor. Quisinostat has proven its efficacy against several tumor types, including melanoma, in pre-clinical studies [16–19] and is currently being tested in phase 2 clinical trials [20, 21]. The antitumor-response observed with HDAC inhibitors is often limited to induction of a G1 cell cycle arrest. Although this effect can block tumor outgrowth [21], finding drugs that can synergize with HDAC inhibitors and promote cancer cell killing would greatly increase their clinical impact. In breast cancer cells HDAC inhibition induced the degradation of cyclin D1 protein, which could implicate that HDAC inhibition would sensitize cells for CDK inhibition [22]. Indeed, in neuroblastoma cell lines HDAC inhibition combined with CDK inhibition induces apoptosis [23].

In this study we aimed at potentiating the effect of quisinostat by combining the treatment with pan-cyclin-dependent kinase (CDK) inhibition using flavopiridol (also known as alvocidib). Flavopiridol is FDA approved and is currently being tested in clinical trials, predominantly as therapeutic intervention for lymphoma and acute myeloid or leukaemia. Flavopiridol strongly inhibits CDK9 activity, but also affects activities of CDK1, CDK2, CDK4, CDK6, CDK7 and CDK12 [24–27]. By inhibiting CDK12, CDK9 and CDK7 flavopiridol inhibits the phosphorylation of serine 2 and 5 within the RNA pol 2 CTD repeats and thereby prevents transcription initiation and elongation [26]. Via the inhibition of CDK1, CDK2, CDK4 and CDK6 flavopiridol induces cell cycle arrests [24, 25]. Interestingly, flavopiridol has been shown to induce stable disease in 7 out of 16 patients with previously untreated metastatic malignant melanoma. Unfortunately, flavopiridol failed to achieve significant clinical benefit according to objective response criteria [28].

Here we show that single treatment with quisinostat or flavopiridol slows down the growth of UM and CM cells, while combined treatment synergistically inhibits growth and, importantly, decreases survival. Whereas single treatment only induced cell cycle arrest, the combination of quisinostat and flavopiridol induced apoptosis of melanoma cells and did so irrespective of their BRAF or NRAS status. Furthermore, melanoma cells with acquired resistance to BRAF inhibition remained as sensitive to the combination as their BRAF sensitive counterparts. The combination also effectively prevented tumor growth *in vivo*, in a patient derived xenograft (PDX) model of CM. In conclusion, we propose that combining quisinostat with flavopiridol should be explored as a first or second line therapeutic option for patients with metastatic UM and CM, respectively.

## RESULTS

### Synergistic reduction of UM cell proliferation by simultaneous CDK and HDAC inhibition

**Figure 1:**
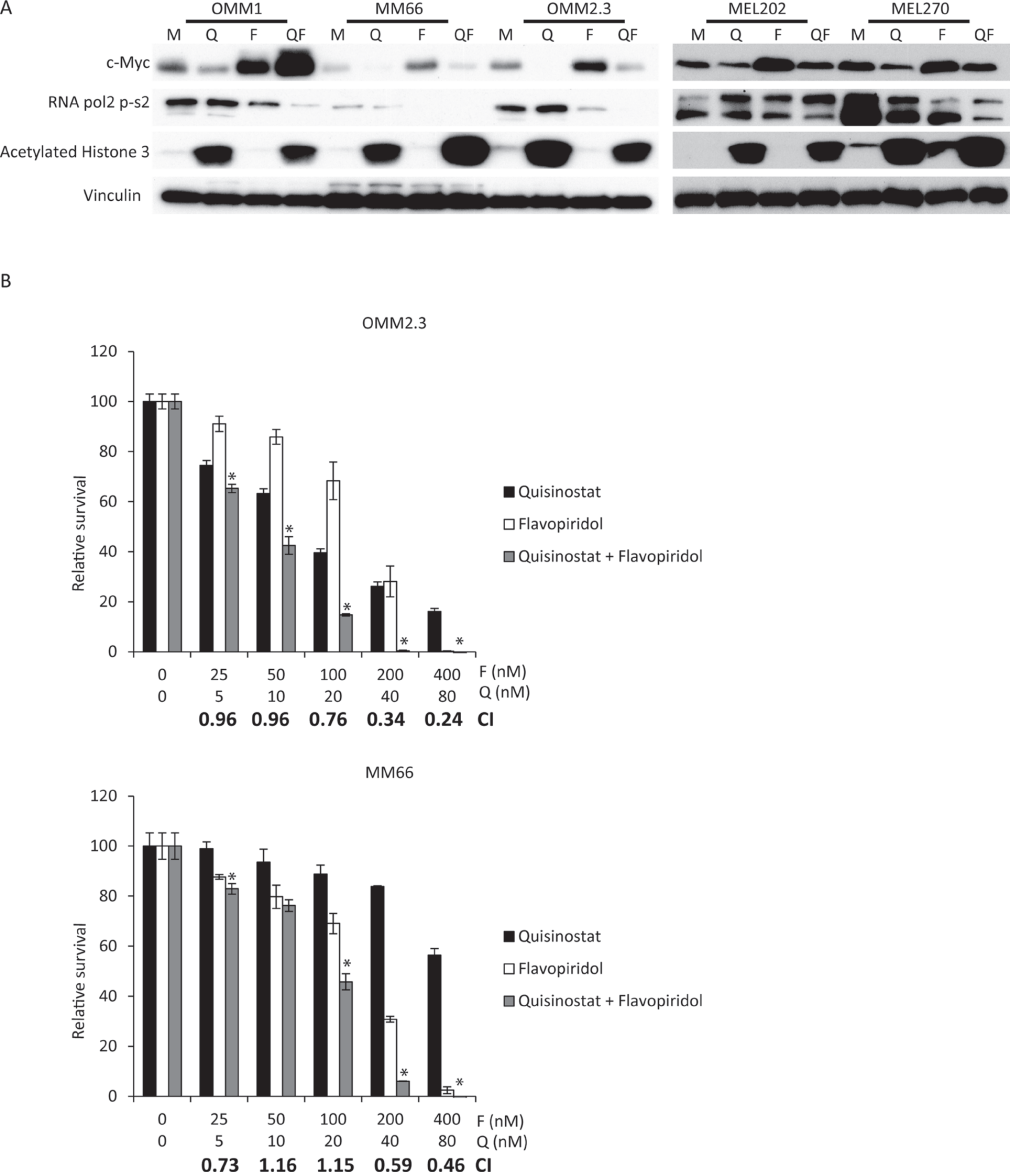
Simultaneous quisinostat and flavopiridol treatment synergistically inhibits growth of UM cell lines (**A**) UM cell lines OMM1, MM66, OMM2.3, MEL202 and MEL270 were treated with 20 nM quisinostat and 100 nM flavopiridol for 24 hours after which cells were harvested. Protein lysates were analyzed for the expression levels of c-Myc, RNA pol2-CTD Ser2 phosphorylation and acetylated histone 3 by Western blot. Expression of vinculin was analyzed to control for equal loading. (**B**) UM cells OMM2.3 and MM66 were treated for 72 hours with indicated concentrations quisinostat and flavopiridol, either alone or in combination to determine effects on cell viability. To determine putative synergism the combination index (CI) values were calculated. Combinations with a significant (*p*: < 0.05) lower relative survival compared to both single treatments are indicated with a^*^.

We first evaluated whether quisinostat and flavopiridol were capable of eliciting their expected biochemical responses in UM cells (Figure 1A). Consistent with quisinostat being an effective inhibitor of HDACs, an increase in acetylation of histone 3 was observed in all UM cell lines exposed to this drug. One of the main targets of flavopiridol is CDK9, which phosphorylates RNA pol2-CTD at Serine 2. Accordingly, reduced phosphorylation of RNA pol2-Ser2 was seen in all but one (MEL202) of the tested UM cell lines exposed to flavopiridol. Counterintuitively, it has been reported that treatment of cells with relatively low concentrations of flavopiridol actually increases the expression of c-Myc at both the RNA and protein level [29]. Indeed, we also find that in all UM cell lines flavopiridol increases c-Myc expression at RNA and protein levels (Figure 1A and Supplementary Figure 1). These data are consistent with flavopiridol being an inhibitor of CDK activity in UM cell lines. The flavopiridol-mediated increase in c-Myc is largely reversed by the addition of quisinostat in most cell lines, as indeed quisinostat in most cases reduces c-Myc levels.

We next examined the effect of quisinostat and/or flavopiridol on UM cell proliferation. In all UM cell lines both quisinostat and flavopiridol reduced relative cell survival in a dose-dependent manner at nanomolar concentrations (Figure 1B and Supplementary Figure 2). Furthermore, a combination of these drugs resulted in an additive (CI: 1.1-0.9) or synergistic (CI: 0.9 >) growth inhibitory effect in all cell lines.

### Cell cycle arrest and apoptosis upon CDK and HDAC inhibition in UM cells

**Figure 2:**
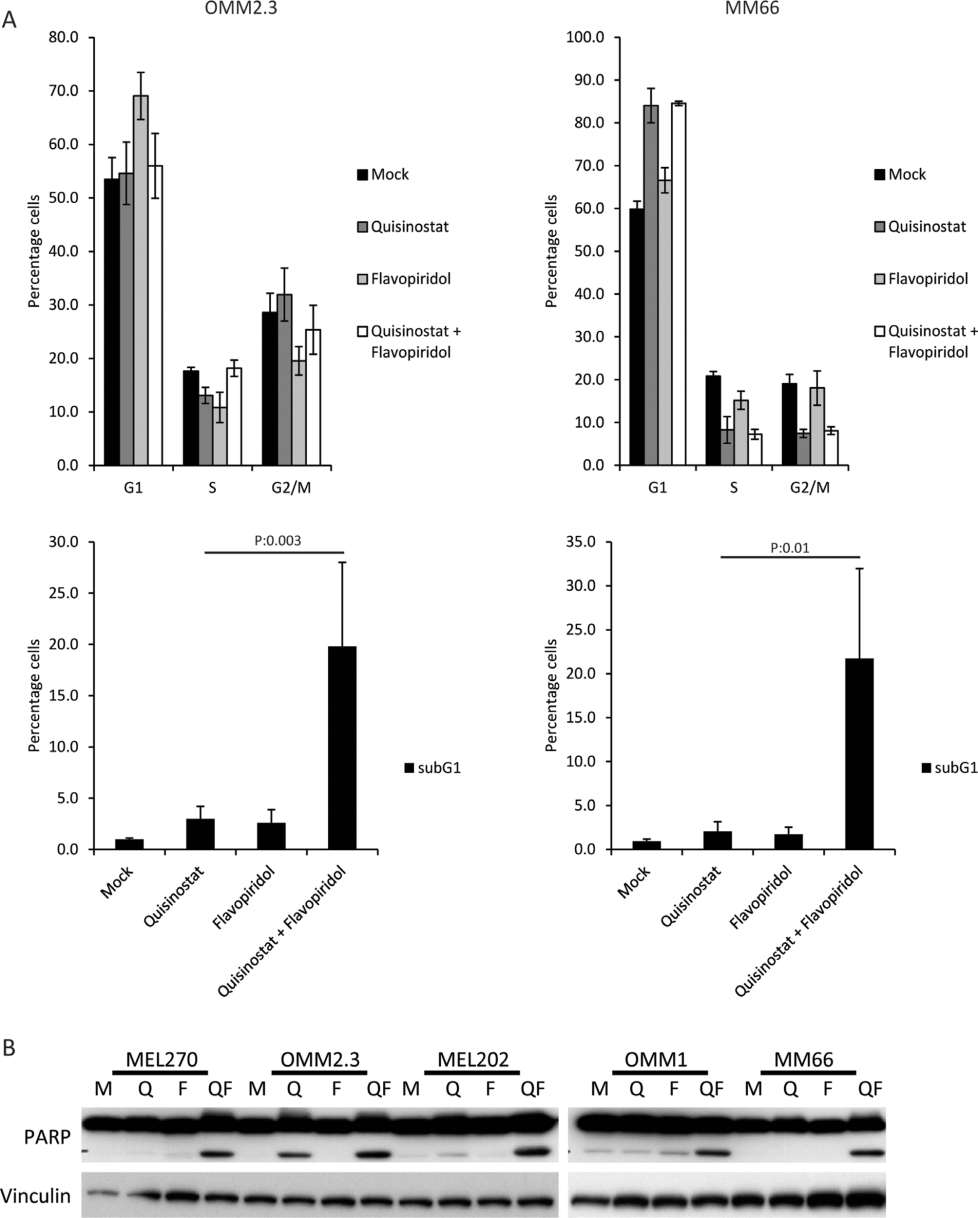
The combination of quisinostat and flavopiridol induces apoptosis in UM cell lines (**A**) OMM2.3 and MM66 cells were treated for 48 hours with 20 nM quisinostat and 100 nM flavopiridol after which cell were harvested to determine the cell cycle profiles by flow cytometry after PI staining. The percentages of each cell cycle phase (G1, S, G2/M and subG1) are the averages of three independent experiments. (**B**) UM cell lines MEL270, OMM2.3, MEL202, OMM1 and MM66 were treated with 20 nM quisinostat and 100 nM flavopiridol for 48 hours. Protein lysates were analyzed by Western blot to investigate PARP cleavage. Expression of vinculin was analyzed to control for equal loading.

Flow cytometry was used to study the effects of the respective drugs on cell cycle progression. In agreement with previous reports, quisinostat induced a G1 cell cycle arrest in MM66, OMM1, MEL202 and MEL270 cells (Figure 2A and Supplementary Figure 3). The increase in G1 population was approximately 20% in these cell lines, concordant with a reduction of both the S- and G2/M- phase populations. However, no G1 arrest was observed upon quisinostat treatment in OMM2.3, although a small decrease in the number of S-phase cells could be observed (Figure 2A). Flavopiridol, due to its ability to inhibit multiple CDKs, has been reported to affect tumor cells at distinct stages during the cell cycle [23, 30]. We observed no obvious changes in the cell cycle profiles of MM66, OMM1 and MEL202 upon flavopiridol treatment, whereas in OMM2.3 cells flavopiridol treatment resulted in a G1 cell cycle arrest and in MEL270 cells in a G2/M cell cycle arrest (Figure 2A and Supplementary Figure 3). In spite of these partly distinct responses to the single compound treatments, the combination of drugs resulted in a significant increase in the subG1 population in all tested UM cell lines, indicating that combined treatment induced cell death (Figure 2 and Supplementary Figure 3). To further explore this increase in subG1, we immunoblotted for PARP. PARP is cleaved by activated caspase 3/7 during apoptosis and can therefore be used as a marker for apoptosis. An increase in cleaved PARP was observed in all cell lines treated with combined quisinostat and flavopiridol (Figure 2B), but not by single treatments. These data show that combining quisinostat and flavopiridol synergistically induce cell death via the induction of apoptosis in UM cell lines.

### Synergistic effects of CDK and HDAC inhibition in cutaneous melanoma cells

**Figure 3:**
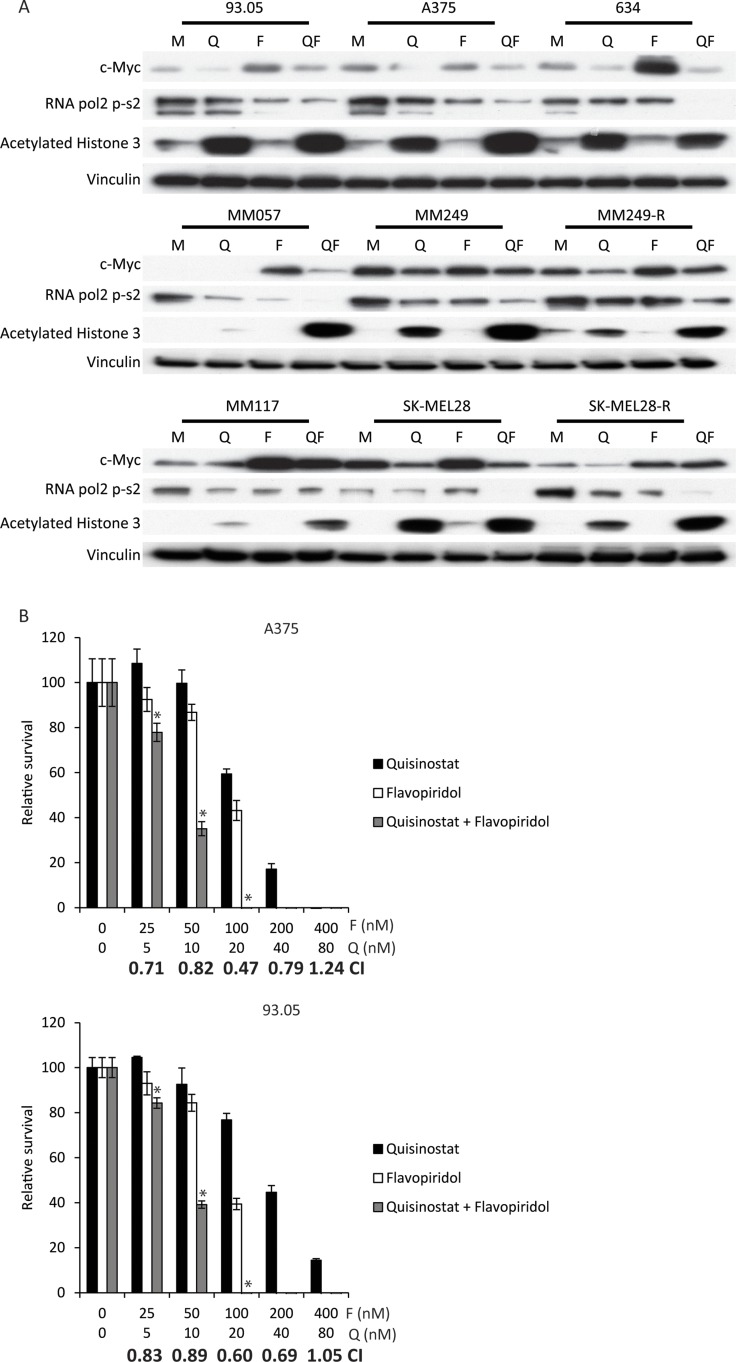
Simultaneous quisinostat and flavopiridol treatment results in synergistic growth inhibition of CM cell lines (**A**) CM cell lines 93.05, A375, 634 (20 nM quisinostat and 100 nM flavopiridol), MM57, SK-MEL28, SK-MEL28R (20 nM quisinostat and 150 nM flavopiridol), MM117, M249 and M249-R (40 nM quisinostat and 200 nM flavopiridol) were treated for 24 hours with indicated concentrations of compounds. Protein lysates were analyzed by Western blotting to investigate levels of c-Myc, RNA pol2-CTD Ser2 phosphorylation and acetylated histone 3. Expression of vinculin was analyzed to control for equal loading. (**B**) A375 and 93.05 cells were treated with quisinostat and/or flavopiridol with indicated concentrations for 72 hours to determine effect on cell viability. To determine putative synergism the combination index (CI) values were calculated. Combinations with a significant (*p*: < 0.05) lower relative survival compared to both single treatments are indicated with a^*^.

Since both quisinostat and flavopiridol are indirectly targeting a plethora of biological processes instead of specific oncogene-driven growth and -proliferation pathways, we explored whether the synergy is uveal specific or could also be observed in cutaneous melanoma (CM). We investigated whether these drugs elicit their biochemical effects in the following BRAF^V600E^ mutated cell lines: 93.05, A375, 634, MM249 and SK-MEL28. Furthermore, NRAS^Q61L^ mutated cell line MM057 and NRAS/BRAF wild-type cell line MM117 were also exposed to these drugs. Treatment with quisinostat increased acetylated histone 3 levels, indicating that quisinostat is efficiently inhibiting HDACs in all cell lines (Figure 3A). Flavopiridol exposure resulted in reduced abundance of RNA pol2-CTD Ser2 phosphorylation in most cell lines but not in 634 and SK-MEL28. c-Myc protein levels were increased upon treatment with flavopiridol in most cell lines. Similar to UM the increase in c-Myc levels was seen at both protein and mRNA levels (Supplementary Figure 1). These data indicate that, like in UM, flavopiridol is actively inhibiting CDKs in CM cell lines. However, the molecular responses upon quisinostat and flavopiridol treatment seemed to vary between cell lines. As observed in UM cell lines, in some CM cell lines concurrent HDAC and CDK inhibition could affect the molecular responses; reversal of flavopiridol induced c-Myc increase, more pronounced drop of RNA pol2-S2 and further increase of acetylated histone 3.

We determined the effect of quisinostat and flavopiridol on the growth/survival of CM cells using cell proliferation assays (Figure 3B and Supplementary Figure 4). The combination of quisinostat and flavopiridol resulted in an additive (CI: 1.1–0.9) or synergistic (CI: 0.9 >) growth inhibitory effect in all CM cell lines tested. Despite the fact that the IC50’s differed per cell line, all IC50’s were in the nanomolar range (Table 1).

**Table 1:** IC50’s for quisinostat and flavopiridol per cell line

cell line	Quisinostat		Flavopiridol	
	IC50 nM	stdev	IC50 nM	stdev
MEL270	5.9	1.8	82.7	14.4
MEL202	24.8	6.4	68.4	10.3
OMM2.3	16.4	1.9	91.3	14.2
OMM1	18.6	2.8	71.3	3.1
MM66	93.0	21.7	99.8	19.3
634	14.8	2.7	133.6	21.0
93.05	36.2	7.3	66.8	7.0
A375	20.8	8.7	65.9	4.5
MM249	23.8	2.0	143.9	6.3
MM249R	17.5	3.1	128.6	24.5
SK-MEL28	30.7	4.4	113.1	6.1
SK-MEL28-R	28.4	6.2	92.6	10.5
MM117	14.8	1.5	178.2	17.8
MM057	66.8	9.5	97.5	18.4

The first line therapy for CM patients carrying the BRAF^V600E^ mutation (∼45% of all patients) consists of concurrent BRAF^V600E^/MEK inhibition or immunotherapy, to which resistance occurs. Therefore, we investigated whether two cell lines that acquired resistance to BRAF inhibition *in vitro*, MM249-R and SK-MEL28-R were still responsive to HDAC/CDK inhibition. Striking responses to both drugs were observed in both the BRAF^V600E^ inhibitor resistant and - sensitive parental cell lines (Figure 3A). Furthermore, the BRAF^V600E^ inhibitor resistant and - sensitive parental cell lines had similar IC50’s for both drugs (Table 1). Importantly, like their parental cell lines, the resistant cell lines showed synergistic or additive CI values upon concurrent treatment with flavopiridol and quisinostat (Supplementary Figure 4).

### Concurrent CDK and HDAC inhibition results in cell cycle arrest and apoptosis in CM cells

**Figure 4:**
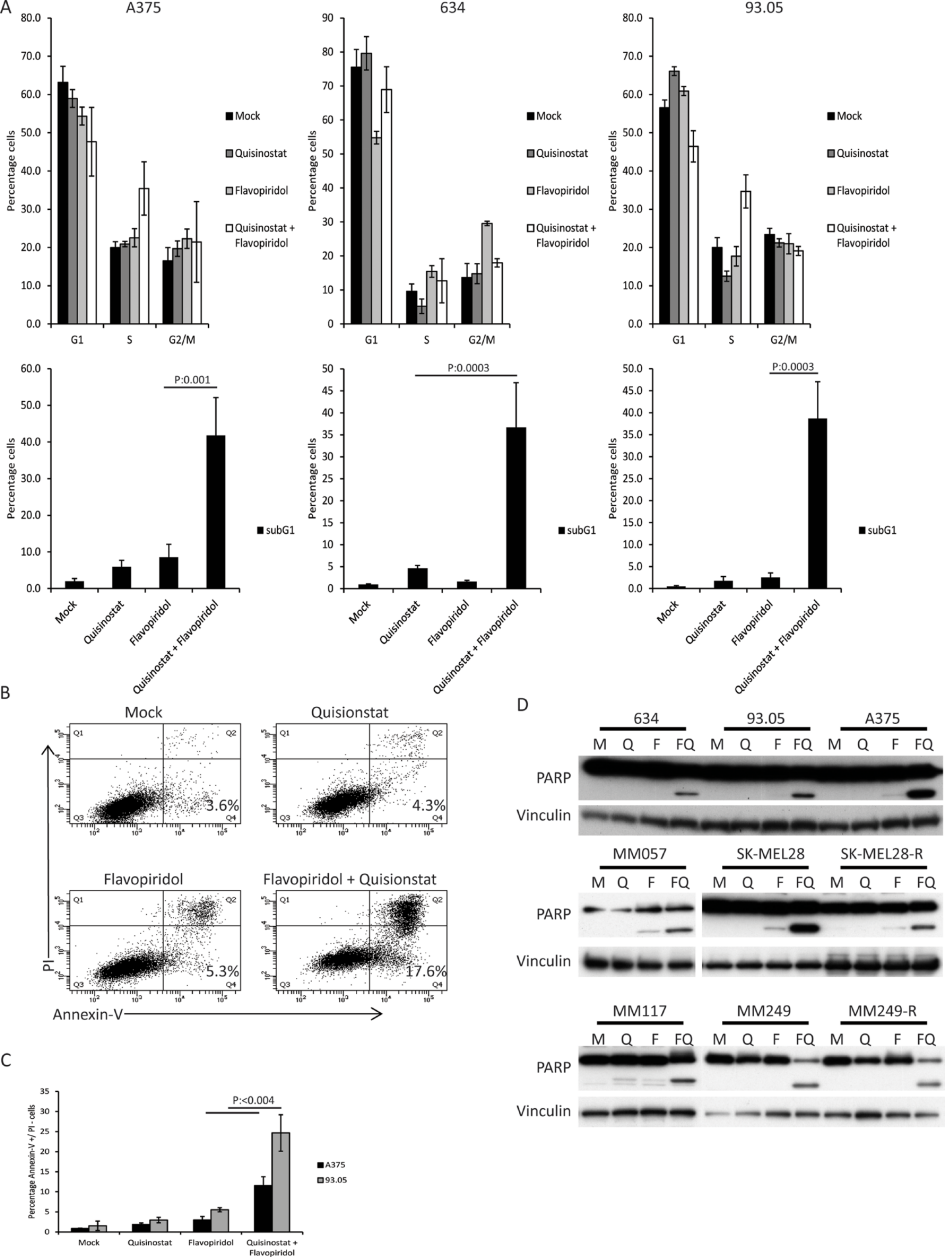
The combination of quisinostat and flavopiridol induces apoptosis in CM cell lines (**A**) A375, 634 and 93.05 were treated with 20 nM quisinostat and 100 nM flavopiridol for 48 hours after which cells were harvested to determine the cell cycle profiles by flow cytometry upon PI staining. The shown percentages of each cell cycle phase (G1, S, G2/M and subG1) are the averages of three independent experiments. (**B**) CM cell lines 93.05, A375, 634 (20 nM Quisinostat and 100 nM Flavopiridol), MM57, SK-MEL28, SK-MEL28-R (20 nM Quisinostat and 150 nM Flavopiridol), MM117, M249 and M249-R (40 nM Quisinostat and 200 nM Flavopiridol) were treated with indicated concentration of drugs for 24 hours. Protein lysates were analyzed by Western blotting to investigate PARP cleavage. Expression of vinculin was analyzed to control for equal loading. (**C**) The percentage of early apoptotic cells was assessed using Annexin V and PI staining, of which a representative experiment is shown using 93.05 cells. (**D**) PI-negative and Annexin V-positive cells were considered to be early apoptotic. Percentages shown are averages of three independent experiments.

To study the mechanism underlying the synergistic growth inhibitory effect observed in response to concurrent inhibition of CDK and HDAC we determined the consequences of quisinostat and flavopiridol exposure on the cell cycle progression of CM cell lines 93.05, 634 and A375 (Figure 4A). Quisinostat induced a minor G1 arrest in 93.05 cells, slightly reduced S-phase in 634 but did not affect A375 cells. Flavopiridol treatment induced a G2/M arrest in 634, but no clear effect in A375 and 93.05. These results show again that different cell lines show distinct responses to quisinostat or flavopiridol treatment. Interestingly, combining both drugs increased the subG1 population in all three cell lines, indicating enhanced cell death (Figure 4A). To study whether this is, at least partly, a consequence of induction of apoptosis, 93.05 and A375 cells were stained with Annexin V-FITC and Propidium Iodide (PI) upon treatment and analyzed by flow cytometry. The results showed that the ‘early’ apoptotic fraction (Annexin V-positive, PI-negative) was increased when quisinostat and flavopiridol were combined (Figure 4B and 4C). To study whether this induction of apoptosis is observed in all different CM cell lines upon combined treatment, PARP cleavage was investigated by immunoblotting. A marked increase in cleaved PARP was evidenced in all cell lines upon quisinostat/flavopiridol exposure (Figure 4D). Given that these cell lines carry different driver mutations, these data show that the induction of apoptosis in response to this combination is independent on the BRAF or NRAS mutational status.

### Concurrent CDK and HDAC inhibition results in growth inhibition *in vivo*

**Figure 5:**
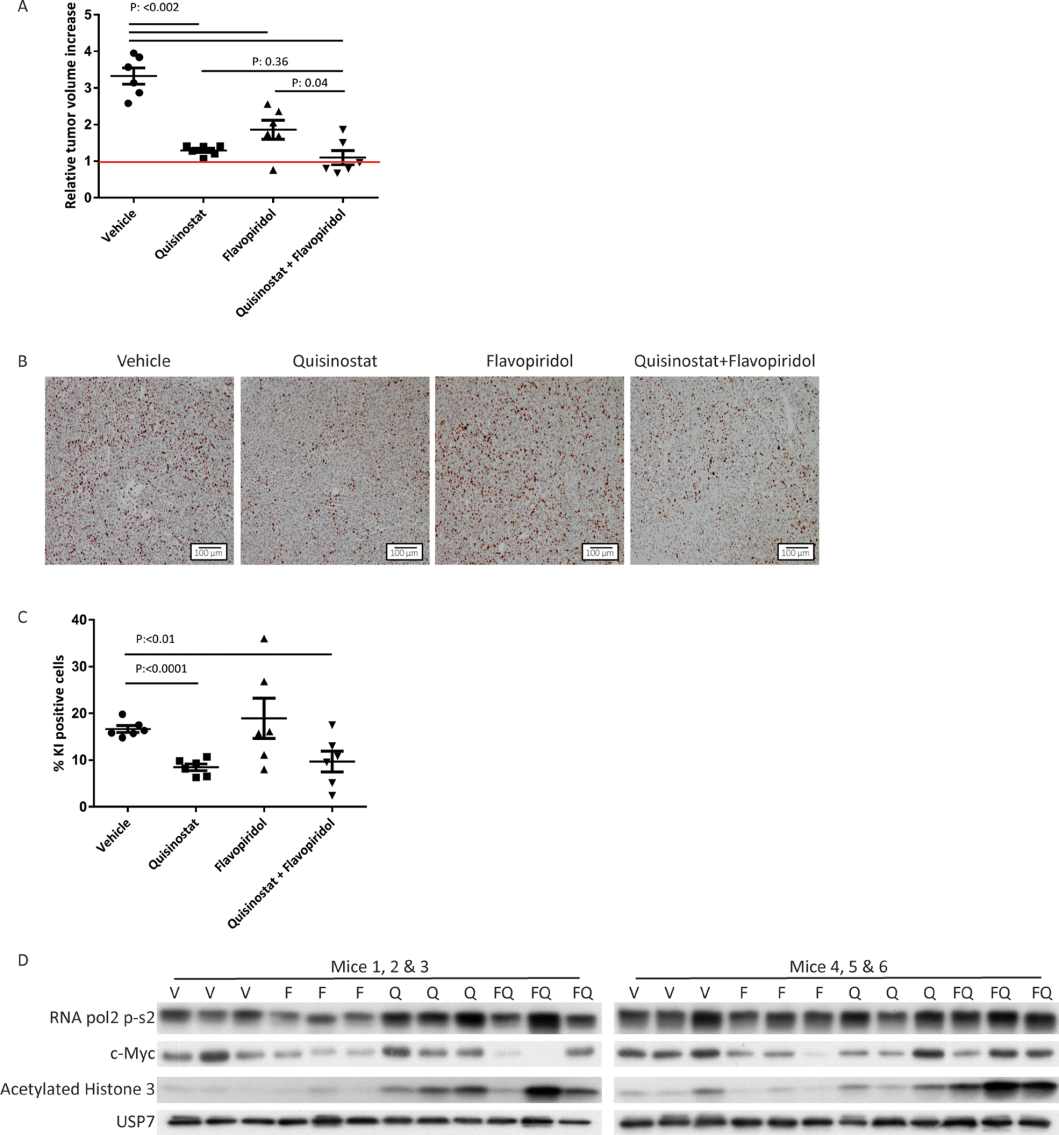
Growth inhibitory and molecular effects of HDAC and CDK inhibition on cutaneous melanoma MEL002 PDX model (**A**) Animals were transplanted with pieces from a patient biopsy. When tumors reached 200 mm^3^ mice were injected intraperitoneally with vehicle, flavopiridol (5 mg/kg), quisinostat (20 mg/kg) or the combination of flavopiridol and quisinostat. Relative tumor increase of the vehicle treated group was on average 3.3-fold, whereas treatment with flavopiridol (5 mg/kg) or quisinostat (20 mg/kg) as single agent resulted in an average tumor increase of 1.9- and 1.3-fold, respectively. Combined therapy resulted in an average tumor increase of 1.1 fold. Out of the six tumors treated with the combination of compounds, three show regression compared to day 0 with a tumor growth of 0.7, 0.8 and 0.8 fold. (**B**) Ki-67 staining was performed to determine the percentage of proliferating cells; representative pictures are shown in. (**C**) Quantification of Ki-67 staining was performed with ImmunoRatio software. (**D**) Protein lysates were analyzed by Western blotting to investigate levels of RNA pol2-CTD Ser2 phosphorylation, c-Myc and acetylated histone 3. Expression of USP7 was analyzed to control for equal loading.

To assess the potential clinical relevance of the quisinostat/flavopiridol combination, we tested its efficacy *in vivo* using a PDX preclinical mouse model of melanoma (MEL002). We used a BRAF wild type cutaneous melanoma tumor as a model as patients with this type of melanoma generally have limited therapeutic options. Once tumors reached a size of 200 mm^2^, drug injections were given intraperitoneally every other day for 28 days. After 28 days, treatment with flavopiridol alone had significantly reduced tumor growth (Figure 5A and Supplementary Figure 5). Quisinostat monotherapy resulted in stable disease. The combined flavopiridol and quisinostat treatment resulted in a decrease in tumor volume significant greater than observed with flavopiridol monotherapy. 3/6 tumors from the combined treatment group showed a slight tumor regression (0.3, 0.2 and 0.2 fold) compared to day 0 (Figure 5A). In agreement with the reduced tumor volume, IHC staining for proliferation marker Ki-67 showed significantly reduced cell proliferation upon quisinostat treatment (Figure 5B and 5C). In flavopiridol treated tumors, either alone or in combination with quisinostat, a strong variation in numbers of Ki-67 positive cells between tumors was observed (Figure 5C), possibly indicating that the tumor growth inhibition is the result of a complex mix of arrests at distinct cell cycle phases.

To evaluate whether quisinostat and flavopiridol affected their respective targets *in vivo* the levels of acetylated histone 3, c-Myc and phosphorylated RNA pol2 CTD were assessed (Figure 5D). We could detect an increase in acetylated histone 3 upon quisinostat treatment, demonstrating the efficacy of quisinostat *in vivo.* Although flavopiridol treatment *in vivo* did not affect RNA pol2-Ser2 phosphorylation or c-Myc protein levels, combination-treated tumors tended to have higher levels of acetylated histone 3, a trend also visible in most *in vitro* treated CM cell lines. Complete histopathological examination of two mice per treatment group showed minimal and moderate toxicity upon treatment (Supplementary Figure 6). Most severe adverse effect found was necrosis of the lymph nodes induced by flavopiridol, which has been described before [31]. Importantly, when these two broad spectrum drugs were combined no increase in severity of the adverse events was found. Suggesting these drugs can be combined in order to enhance clinical benefits, without enhancing adverse effects.

## DISCUSSION

Despite recent advancements in the clinic, both metastasized uveal and cutaneous melanomas remain difficult to cure. For CM, advances have been made with respect to the optimization of mutated BRAF-targeting therapies [4], with or without MEK inhibitors, and immunotherapy has made it in some cases to first-line treatment [5]. Even so, a large proportion of CM patients does not respond to these therapies or eventually develop resistance. For metastasized UM no effective treatment is available in the clinic [32, 33].

To find a novel general therapeutic intervention for most, if not all, melanoma patients, we focused on compounds targeting pathways broadly deregulated in most cancer cells. This study focusses on the HDAC inhibitor quisinostat and the CDK inhibitor flavopiridol, both currently in clinical trials for various types of cancer. This implicates that promising pre-clinical results with these compounds can be implemented in the clinic relatively quickly, as toxicity of both single agents has already been assessed.

Our results show that, in agreement with previous studies, both the HDAC inhibitor quisinostat and the CDK inhibitor flavopiridol exert their respective anticancer functions independent of the type of driver mutations [16, 17, 28, 30, 34]. Quisinostat induces a G1 cell cycle arrest in tested UM cell lines, consistent with previous published results from Landreville *et al.* [12]. Despite the ability of quisinostat to inhibit HDACs in both CM and UM cell lines, our results suggest that CM and UM cell lines respond partly distinct to this compound. Whereas 80% (4/5) of tested UM cell lines show a G1 cell cycle arrest, only 1 out of 3 CM cell lines (BRAF mutant) tested showed only a modest increase (10%) in the G1 population. Differences in response to quisinostat can be attributed to potential differences in expression of various HDACs or variation in other effector protein expression. Regardless of the differences in mechanism of action of quisinostat between these different cell lines, it appears that all cell lines are growth inhibited by quisinostat with IC50s in the low nanomolar range.

According to previous studies the anticancer effects of flavopiridol are even more widespread, due to its ability to inhibit multiple CDK’s, hampering both transcription (by inhibition of CDK9, CDK12 and CDK7) and the cell cycle, at multiple phases (via the inhibition of CDK1, CDK1, CDK4 and CDK6) [27, 29, 30, 34]. Apart from these well described targets, it has been reported recently that flavopiridol inhibits glycogen phosphorylase, reducing the available glucose for glycolysis of cancer cells [35]. Succeeding this report, it has been demonstrated that flavopiridol reduces various components of the glycolytic pathway in glioblastoma cell lines, limiting glycolysis, which could be a new perspective to flavopiridol [36]. Despite these broad ranges of molecular effects by flavopiridol, the drug is well tolerated in patients while inducing tumor regression [37]. Regardless of these wide-spread effects, nearly all melanoma cell lines responded similar to flavopiridol treatment at a molecular level, i.e. the reduction of RNA pol2 CTD Ser2 phosphorylation and the increase in c-Myc protein levels. The increase in c-Myc was mediated by enhanced gene transcription, rather than post transcriptional regulation, which is associated with low concentrations of flavopiridol [29]. In combination with quisinostat, these low flavopiridol concentrations have synergistic effects via the induction of apoptosis, potentially reducing adverse effects. Although the underlying mechanism of the induction of apoptosis remains elusive it could be hypothesized that both drugs influence each other in a positive manner; for example, the observed further reduction of RNA pol2 CTD phosphorylation in the presence of quisinostat. Based upon literature showing that both CDK9 and HDAC inhibition decrease expression of the anti-apoptotic protein MCL-1 and thereby stimulate apoptosis [18, 38–43] one could propose that the combination treatment further reduces MCL-1 levels. It must be noted that concentrations of flavopiridol used to achieve these effects on MCL-1 expression tend to be in the micromolar range whereas in this study cells were exposed to flavopiridol in a nanomolar range. Probably therefore we could not detect consistent changes in MCL-1 levels using our experimental design (data not shown). However, it could be that MCL-1 will play an important role when high concentrations are used in a more (pre-) clinical setting. Similarly, expression levels of other Bcl-2 family members reported to be affected by quisinostat and/or flavopiridol were not significantly or consistently affected under our experimental settings.

In our study combined flavopiridol and quisinostat treatment significantly reduced tumor growth in a cutaneous melanoma PDX model. Quisinostat increased the level of acetylated histone 3 concomitant with a strongly reduced tumor cell proliferation. Strikingly, in the tumors treated with flavopiridol, either alone or in combination with quisinostat, the number of Ki-67 positive cells is highly variable, possibly indicating that the growth retardation induced by flavopiridol is a complex mixture of arrests at various cell cycle phases as discussed above. At a molecular level we could not confirm activity of flavopiridol in the treated tumors, although dose and regimen was comparable to previous studies [44, 45]. This could implicate that the molecular effects of flavopiridol are more transiently *in vivo* compared to *in vitro*, possibly caused by clearance of flavopiridol from the body, which only takes hours in humans [46]. Treatment with flavopiridol did inhibit the tumor growth and resulted in tumor regression in 50% of the mice treated with both quisinostat and flavopiridol. Interestingly, these beneficial effects could be achieved without enhancing adverse effects induced by these two broad spectrum drugs. In order to achieve similar synergistic effects *in vivo* compared to *in vitro,* our data suggest that a different treatment regime and/or dosage of flavopiridol should be used. Based on the results presented in this study it could be hypothesized that increasing the effect of flavopiridol could potentially synergistically enhance the effects of quisinostat, possibly resulting in tumor regression *in vivo*.

In conclusion, our data show that the combination of quisinostat and flavopiridol treatment inhibits melanoma cell viability synergistically by inducing apoptosis, independent of driver mutations and acquired BRAF inhibitor resistance. Simultaneous HDAC and CDK inhibition could be a potential therapeutic intervention for those melanoma patients that have relapsed on BRAFi treatment, since BRAFi-sensitive and BRAFi-resistant cell lines respond equally effective to this combination of compounds. It seems unlikely that one mutation or epigenetic change is able to induce resistance to this combination, since quisinostat and flavopiridol inhibits multiple HDACs and CDKs. Therefore, we propose this novel therapeutic intervention as treatment option for patients with metastasized UM. Moreover, combined quisinostat/flavopiridol treatment could be used as first-line treatment in CM patients that have a BRAF wild type tumor. Lastly, since the combination treatment has shown promising results in BRAF inhibitor-resistant cells, also relapsed patients under BRAF inhibitor treatment could benefit from our optimized combinatorial treatment regimen. This treatment could be implemented in the clinic rather easily since both quisinostat and flavopiridol are already in clinical trials.

## MATERIALS AND METHODS

### Cell culture

The UM cell lines MEL270, MEL202, OMM2.3 and OMM1 were cultured in a mixture of RPMI and DMEM-F12 (1:1 ratio), supplemented with 10% fetal calf serum (FCS) and antibiotics. OMM1 was provided by Gré Luyten (LUMC, Leiden, The Netherlands) and MEL270, MEL202, OMM2.3 were a kind gift of Bruce Ksander (Schepens Eye Research Institute, Boston, MA, USA). Establishment of the UM cell line MM66 has been described [47], was kindly provided by Sergio Roman-Roman (Curie Institute, Paris, France) and were cultured in IMDM containing 20% FCS and antibiotics. The CM cell lines A375, 634 and 93.05 were cultured in DMEM/high glucose supplemented with 10% FCS and antibiotics. M117 and M057 CM cell lines were cultured in DMEM-F10 with 8% FCS. SK-MEL28 was maintained in RPMI plus 10% FCS plus antibiotics. DMEM/high glucose containing 5% FCS/antibiotics was used to maintain the M249 CM cells. Medium for the BRAF inhibition resistant derivatives of SK-MEL28 and M249 was supplemented with 1µM PLX-4032 (Selleck Chemicals, Houston, TX, USA). All cell lines were cultured for no more than 20 passages after thawing and were checked regularly for mycoplasma.

### Western blot analysis

Cells were rinsed twice with ice-cold PBS and lysed in Giordano buffer (50 mM Tris-HCl pH7.4, 250 mM NaCl, 0.1% Triton X-100 and 5 mM EDTA; supplemented with phosphatase- and protease inhibitors). Equal protein amounts were separated using SDS-PAGE and blotted onto polyvinylidene fluoride transfer membranes (Millipore, Darmstadt, Germany). After blocking the membranes in TBST (10 mM Tris-HCl pH8.0, 150 mM NaCl, 0.2% Tween 20) containing 10% milk, membranes were incubated with the proper primary antibodies (listed in Table 2) and appropriate HRP-conjugated secondary antibodies (Jackson Laboratories, Bar Harbor, ME, USA). Bands were visualized using chemoluminescence and visualized by exposure to X-ray film.

**Table 2:** List of antibodies used for western blot

Protein	Name/Cat^#^	Company
Vinculin	hVIN-1/ V9131	Sigma-Aldrich
PARP	9542	Cell Signaling Technology
RNA pol2 p-S2	AB5095	Abcam
c-Myc	AB32072	Abcam
Acetylated histone 3	31994	Millipore

### Cell growth and viability assays and calculation of synergism

Cells were seeded in triplicate, in 96-well format and incubated for 72 hours. Cell survival was determined via the CellTitre-Blue Cell Viability assay (Promega, Fitchburg, WI, USA); the fluorescence was measured in a microplate reader (Victor, Perkin Elmer, San Jose, CA, USA). Synergism between flavopiridol and quisinostat was calculated using Compusyn software (Paramus, NJ, USA). Combination Index (CI) values below 0.9 were considered to be synergistic, between 0.9 and 1.1 additive effects and above 1.1 to be antagonistic. Flavopiridol was obtained from Selleck Chemicals (Houston, TX, USA) and Quisinostat was kindly provided by Johnson & Johnson.

### Flow cytometry

For cell cycle analysis the cells were harvested by trypsinization, washed twice in PBS and fixed in ice cold 70% ethanol. After fixation, cells were washed in PBS containing 2% FCS and resuspended in PBS containing 2% FCS, 50 µg/ml RNAse and 50 µg/ml propidium iodide (PI). Flow cytometry analysis was performed using the BD LSR II system (BD Biosciences, San Diego, CA, USA). To determine presence of apoptotic cells by Annexin V staining, cells were harvested and washed twice in PBS, resuspended in Annexin V-binding buffer in presence of FITC-labelled Annexin V (Sigma-Aldrich, Saint Louis, MO, USA) and PI, following incubation for 10 minutes at room temperature. Cells staining negative for PI, but positive for Annexin V were considered to be apoptotic. Cells staining positive for both PI and Annexin V were considered to be late apoptotic or necrotic and, therefore, excluded from the analysis.

### RNA isolation, cDNA synthesis and real-time quantitative PCR

RNA was isolated using the SV total RNA isolation kit (Promega), after which cDNA was synthesized using the reverse transcriptase reaction mixture as indicated by Promega. qPCR was performed using SYBR green mix (Roche Diagnostics, Indianapolis, IN, USA) in a C1000 touch Thermal Cycler (Bio-Rad laboratories, Hercules, CA, USA). In three independent experiments relative expression of c-Myc (fw: GCCACGTCTCCACACATCAG, rev: TGGTGCATTTTCGGTTGTTG), compared to housekeeping genes CAPNS1 (fw: ATGGTTTTGGCATTGACACATG, rev: GCTTGCCTGTGGTGTCGC) and SRPR (fw: CATTGCTTTTGCACGTAACCAA, rev: ATTGTCTTGCATGCGGCC) was determined. Average relative expression per experiment was compared to the untreated set at 1.

### Patient derived xenograft mouse model

Tumor pieces of cutaneous melanoma tumor model MEL002 (BRAF wild type) were transplanted interscapular in NMRI nude mice as described by M. Dewaele *et al.* [48]. When tumor volume reached 200 mm^3^ 6 animals per group were treated intraperitoneally, with either vehicle, quisinostat (20 mg/kg), flavopiridol (5 mg/kg) or the combination every other day for 28 days. Bodyweight was measured to monitor the animals. During the treatment tumor volume was assessed every other day using a caliper and calculated (tumor volume mm^3^ = (width^2^ × length)/2). At the end of the experiment all animals were sacrificed and tumors were removed, general necropsy was performed on 2 mice per group. Immunohistochemical (IHC) staining to asses tumor cell proliferation were performed as described by Hawinkels *et al.* [49] using primary antibody Ki-67 1:500 diluted (AB9260, Millipore). Three to five representative pictures were taken per tumor of which an average percentage of Ki-67 positive cells was determined per tumor using the ImmunoRatio web application as described by Tuominen *et al.* [50]. Tumor pieces were lyzed using the TissueLyser LT (Quiagen, Hilden, Germany) according to manufacturer’s protocol in RIPA lysis buffer (150 mM NaCl, 1% NP-40, 0.25% deoxycholate, 0.1% SDS, 50 mM Tris-HCl pH 8.0, 2 mM EDTA; supplemented with phosphatase- and protease inhibitors) followed by western blot analysis, as described above.

### Statistical analysis

Differences between two groups were calculated using Student’s *t*-test. To determine the difference in tumor growth over time between groups in the PDX model a two way ANOVA was used. *P*-values of < 0.05 were considered to be significant.

## SUPPLEMENTARY MATERIALS FIGURES


