# Supplementary material for: Combined inhibition of CDK and HDAC as a promising therapeutic strategy for both cutaneous and uveal metastatic melanoma
Source: Oncotarget. 2017 Dec 15;9(5):6174–87. doi: 10.18632/oncotarget.23485 (PMC5814203; doi:10.18632/oncotarget.23485)
Supplement: Supplementary file 1 [file oncotarget-09-6174-s001.pdf]

Combined inhibition of CDK and HDAC as a promising therapeutic strategy for both cutaneous and uveal metastatic melanoma

SUPPLEMENTARY MATERIALS

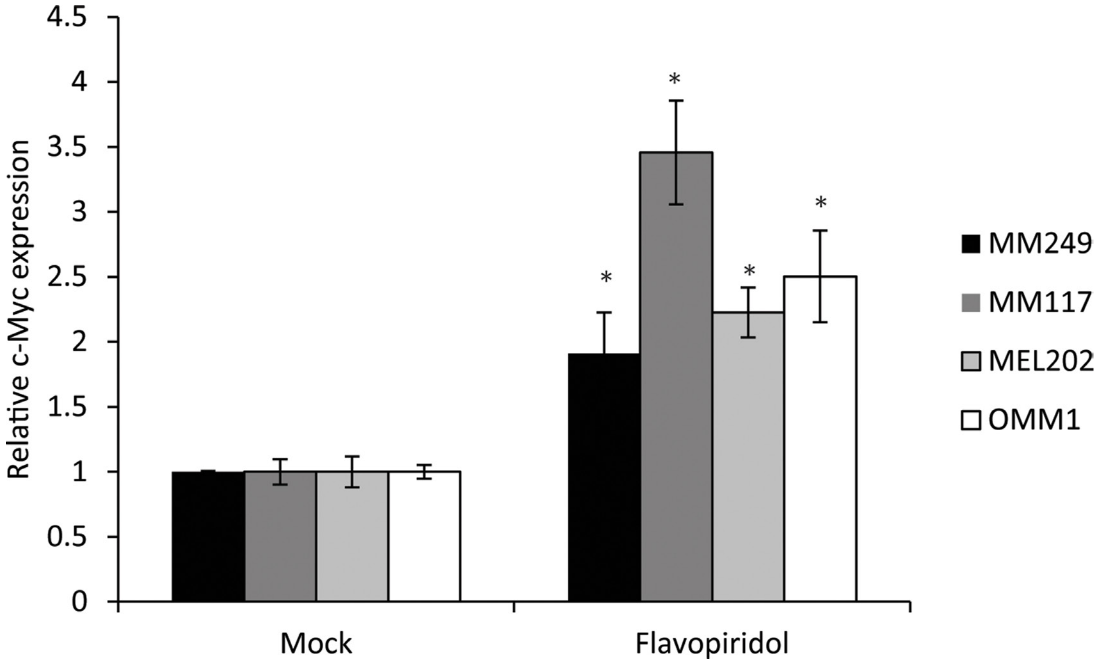

Supplementary Figure 1: Increase in c-Myc mRNA expression upon flavopiridol treatment. CM cells MM249 and MM117 and UM cells MEL202 and OMM1 were treated for 8 hours with flavopiridol (200, 150, 100 and 100 nM, respectively). Cells were harvested, RNA isolated, cDNA was synthesized and expression of c-Myc mRNA was determined. Relative expression compared to untreated controls is plotted, when the difference was found to be significant (p: < 0.05) compared to the control this was indicated with a*.

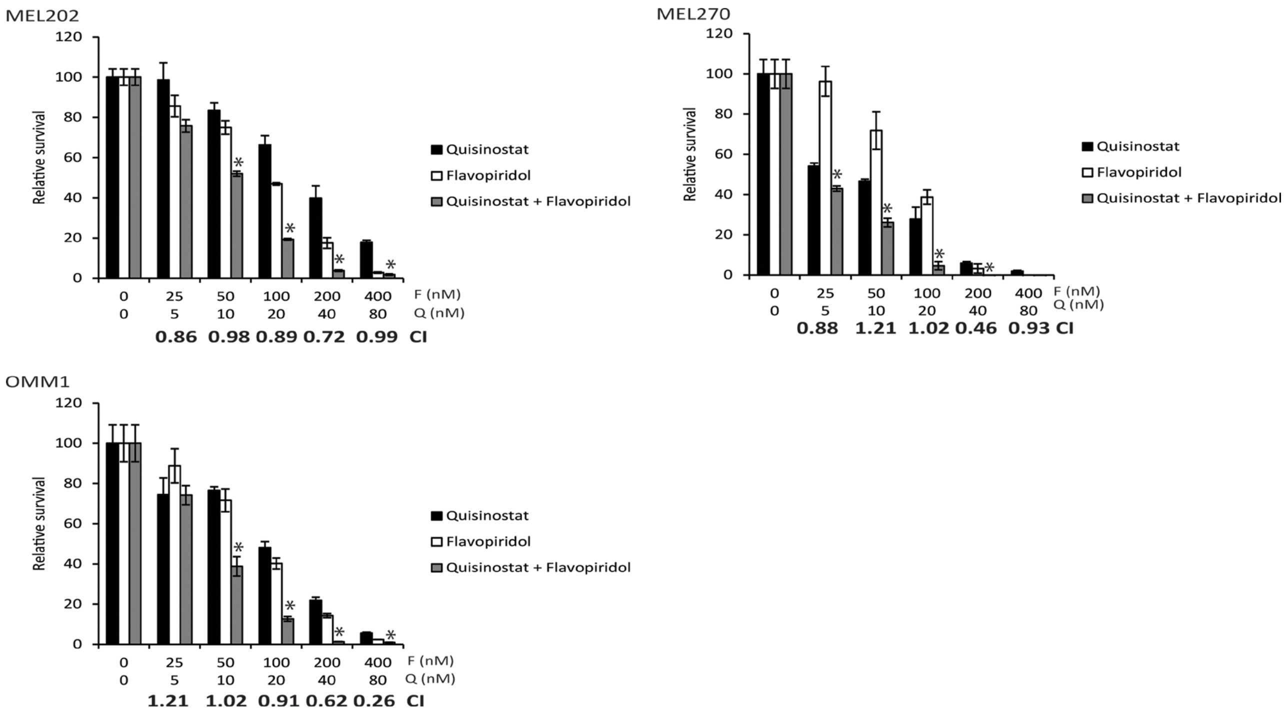

Supplementary Figure 2: Synergistic growth inhibition of CDK and HDAC inhibition on UM cells. UM cells were treated with the indicated concentrations of flavopiridol and quisinostat, alone or in combination. After 72 h the cell viability was determined. To determine the extent of synergism the combination index (CI) was used. Combinations with a significant (p: < 0.05) lower relative survival compared to both single treatments are indicated with a*.

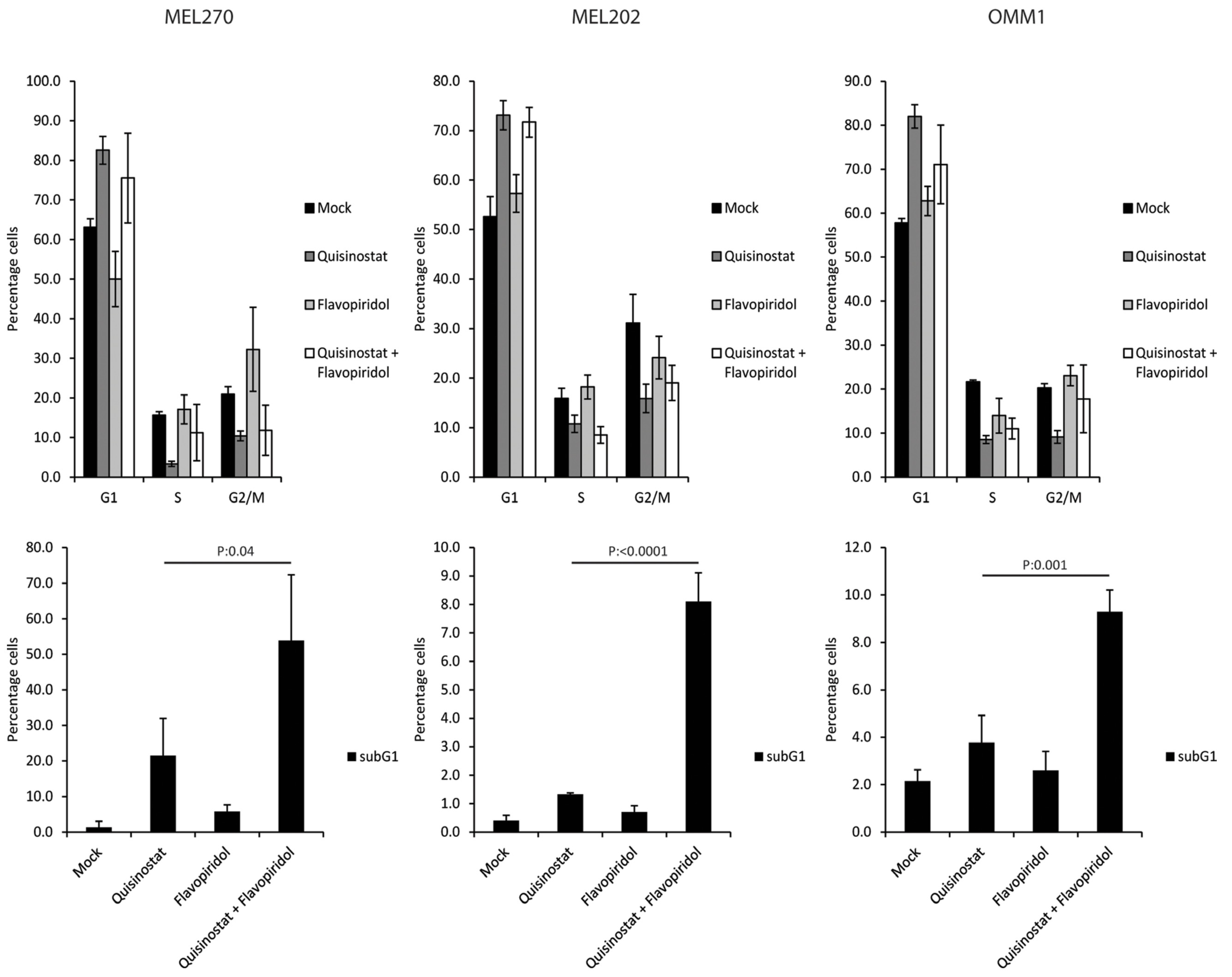

Supplementary Figure 3: Effect of CDK and HDAC inhibition on the cell cycle progression of uveal melanoma cells. MEL270, MEL202 and OMM1 cells were treated for 48 hours with 20 nM quisinostat and 100 nM flavopiridol after which cells were harvested to determine the cell cycle profiles by flow cytometry upon PI staining. The shown percentages of each cell cycle phase (G1, S, G2/M and subG1) are averages of three independent experiments.

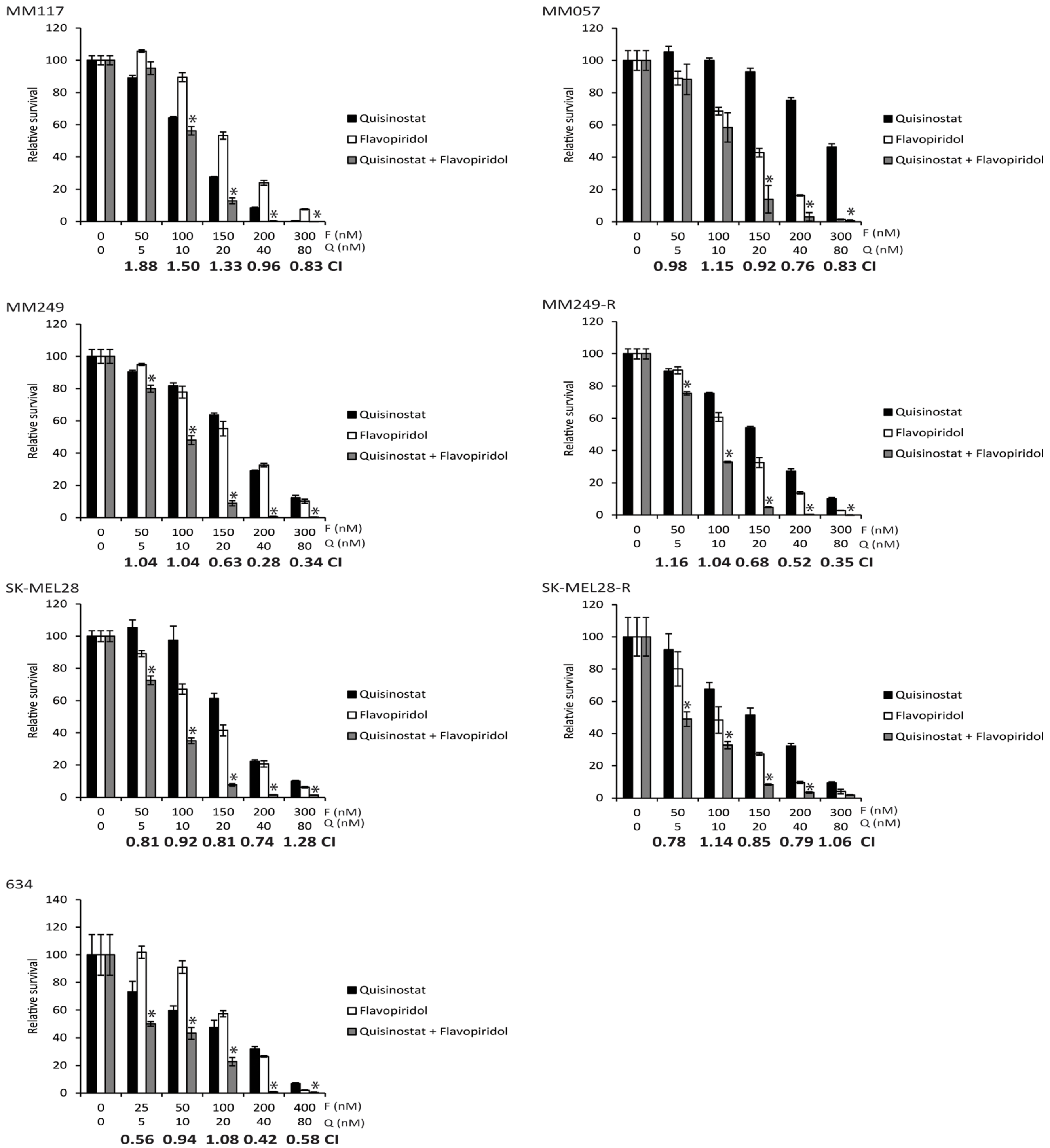

Supplementary Figure 4: Synergistic growth inhibition of CDK and HDAC inhibition on CM cells. CM cells were treated with the indicated concentrations of flavopiridol and quisinostat, alone or in combination. After 72 h the cell viability was determined. To determine the extent of synergism the combination index (CI) was used. Combinations with a significant (p: < 0.05) lower relative survival compared to both single treatments are indicated with a*.

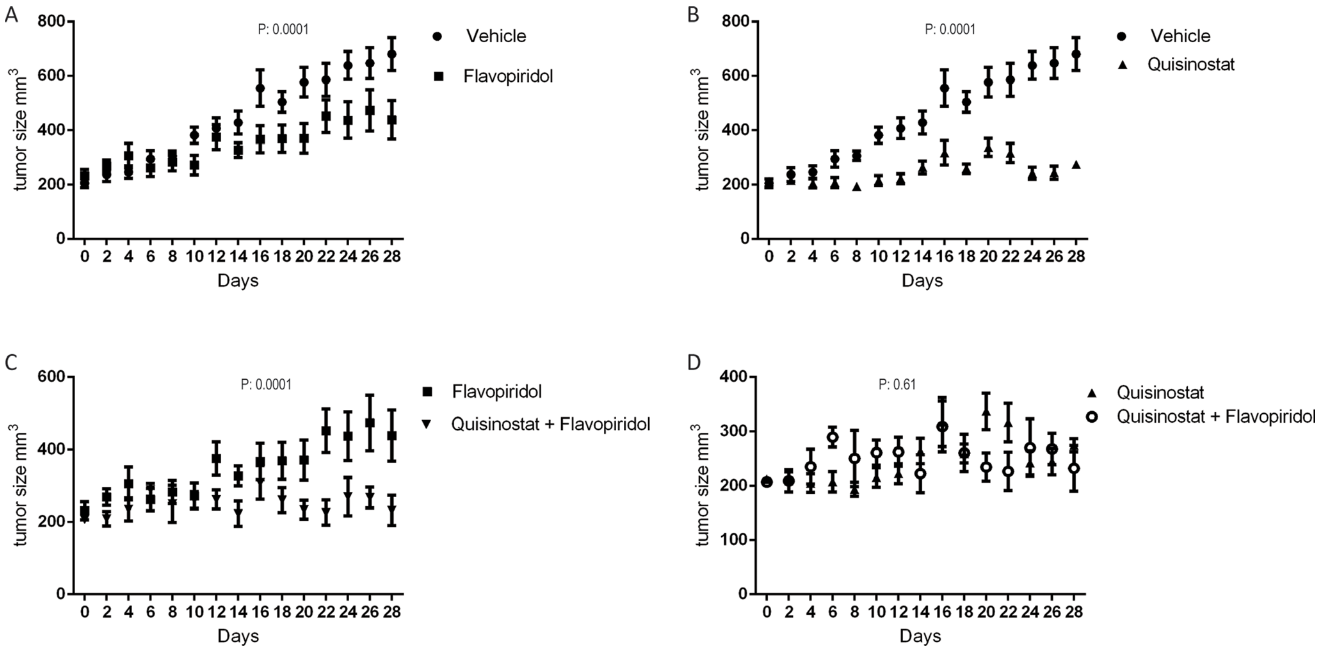

Supplementary Figure 5: Growth inhibitory effect of HDAC and CDK inhibition on MEL002 PDX model in time. Animals were transplanted with tumor pieces. When tumors reached 200 mm^3 mice were injected I.P. every other day for 28 days. (**A**) Mice were treated with flavopiridol (5 mg/kg), (**B**) quisinostat (20 mg/kg) or (**C**) Mice treated with flavopiridol compared to combination treated mice. (**D**) Mice treated with quisinostat compared to combination treated mice. Tumor volume was assessed by caliper.

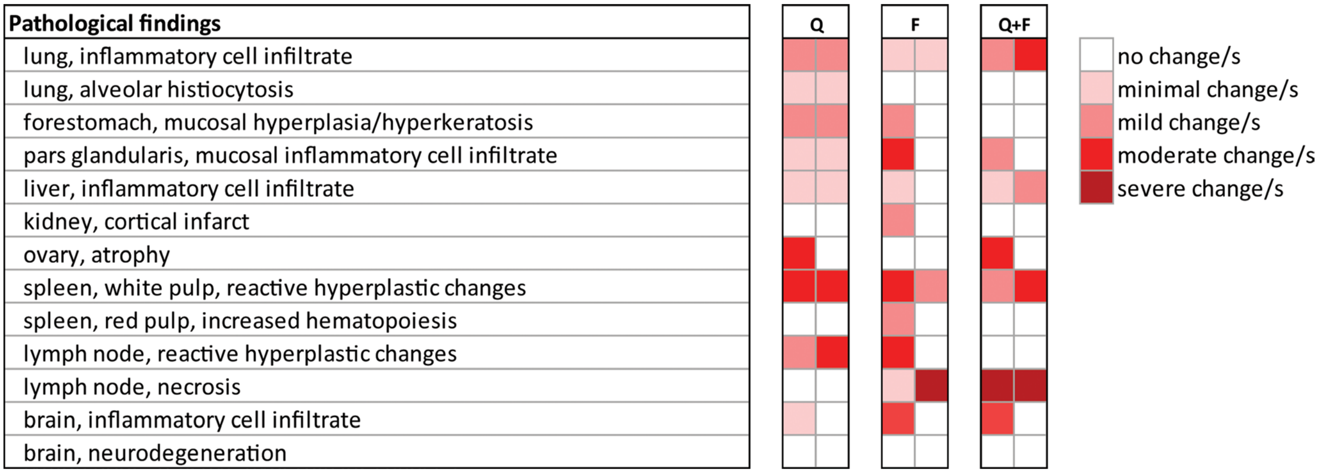

Supplementary Figure 6: Lesion heat map of histopathological examination. Complete histopathological examination was performed on two animals treated with either quisinostat (Q), flavopiridol (F) or both (Q + F).
